# Periodontopathic Microbiota and Atherosclerosis: Roles of TLR-Mediated Inflammation Response

**DOI:** 10.1155/2022/9611362

**Published:** 2022-03-07

**Authors:** Yang Zou, Yaowei Huang, Siqin Liu, Juan Yang, Wenxia Zheng, Yiting Deng, Miaoyu Zhang, Zhenxing Yan, Huifang Xie

**Affiliations:** ^1^Department of Neurology, Zhujiang Hospital, Southern Medical University, 253 Gongye Avenue Middle, Guangzhou City, Guangdong Province 510282, China; ^2^Department of Neurology, Nanfang Hospital, Southern Medical University, 1838 North Guangzhou Avenue, Baiyun District, Guangzhou City, Guangdong Province 510515, China; ^3^Center for Stroke Berlin, Berlin 13353, Germany; ^4^Department of Neurology, Shunde Hospital, Southern Medical University, No. 1, Jiazi Road, Lunjiao Town, Shunde District, Foshan, Guangdon Province 528308, China

## Abstract

Atherosclerosis is a chronic inflammatory disease with a high prevalence worldwide, contributing to a series of adverse cardiovascular and cerebrovascular diseases. Periodontal disease induced by pathogenic periodontal microbiota has been well established as an independent factor of atherosclerosis. Periodontal microorganisms have been detected in atherosclerotic plaques. The high-risk microbiota dwelling in the subgingival pocket can stimulate local and systematic host immune responses and inflammatory cascade reactions through various signaling pathways, resulting in the development and progression of atherosclerosis. One often-discussed pathway is the Toll-like receptor-nuclear factor-*κ*B (TLR-NF-*κ*B) signaling pathway that plays a central role in the transduction of inflammatory mediators and the release of proinflammatory cytokines. This narrative review is aimed at summarizing and updating the latest literature on the association between periodontopathic microbiota and atherosclerosis and providing possible therapeutic ideas for clinicians regarding atherosclerosis prevention and treatment.

## 1. Introduction

Periodontitis is a chronic inflammatory disease with the destruction of connective tissue and alveolar bone that support the teeth [1]. It is induced by pathogenic periodontal microbiota. Up to 42.2% of US dentate adults aged ≥30 years undergone periodontitis [2]. Increasingly, studies support that there is a close association between periodontitis and systematic diseases [3].

Atherosclerosis is a chronic inflammatory disease of the arterial vessel wall and highly prevalent among the general population. Inflammation has a critical role in the atherosclerosis [4]. Atherosclerosis can lead to a series of adverse arteriosclerotic vascular disease (ASVD) [5], including cardiovascular disease (CVD) [6], cerebrovascular disease (CBVD) [7], and peripheral artery disease (PAD) [8]. A report from the American Heart Association shows that CVD is the primary cause of death worldwide, and the mortality will continue to increase to approximately 23.6 million by 2030 [9].

**Figure 1:**
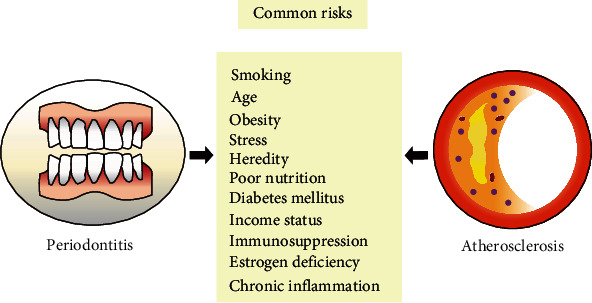
Common risk factors between periodontitis and atherosclerosis.

Periodontitis and atherosclerosis are both the social-economic burden worldwide and have shared common risk factors (Figure 1), such as age, heredity, smoking, diabetes mellitus, hypertension, estrogen deficiency in women, a low socioeconomic status, and stress [10]. Large cohort and epidemiology studies have demonstrated that periodontal infection increased the risk of atherosclerotic cardiovascular and cerebrovascular disease [11–13]. Meanwhile, alterations in the periodontal microbiota of atherosclerotic cohorts and animal models indicated that periodontopathic microbiota might exert a certain effect on the pathogenesis of atherosclerosis. The mechanisms of periodontopathic microbiota-mediated atherosclerosis included inflammation, dyslipidemia, and endothelial dysfunction [14, 15]. Toll-like receptors (TLRs) are the most characteristic members of pattern recognition receptors (PRRs). TLRs are located on the cell membrane or in the cytoplasm and played a critical role on recognizing pathogens and triggering inflammation, contributing to the development of atherosclerosis [16, 17]. Thus, this study is aimed at systematically overviewing the implications of periodontal microbiota on cerebral atherosclerosis and how TLRs were involved in this process.

## 2. Links of Periodontal Infection and Atherosclerotic Cerebrovascular Diseases Based on Clinical and Experimental Evidence

Previous studies pointed that the infection from gut, respiratory, and oral cavity may be responsible for the pathogenesis of atherosclerosis [18]. In 2000, Wu et al. [19] reported that a 2.11-fold increased risk for cerebrovascular accidents was observed with periodontal diseases by utilizing the data of 9962 adults aged 25 to 74 years from the First National Health and Nutrition Examination Survey (NHANES I) and its Epidemiologic Follow-up Study (NHEFS). In 2003, Desvarieux et al. [11] conducted the oral infection and vascular disease epidemiology study (INVEST), which enroll 711 adults without history of cardiovascular and cerebrovascular diseases; there was a linear relationship between the incidence of carotid plaque and the severity of oral infections. Compared to the patients with missing 0 to 9 teeth, a higher prevalence of carotid plaque existed in patients with missing 10 to 19 teeth. Boillot et al. [20] further illustrated whether a connection exists between the activity of inflammatory enzymes from periodontal microbiota and vascular diseases. A prospective cohort study (INVEST) which totally enrolled 593 dentate adults (age 68.7 ± 8.6 years) was performed to demonstrate that increased level of secretory phospholipase A2 (s-PLA2) was associated with higher periodontal bacterial etiologic dominance. Another longitudinal cohort study performed by Schulz et al. [6] detected 11 periodontal pathogens and involved 1002 patients with cardiovascular diseases and identified that the *Eikenella corrodens*, a Gram-negative opportunistic bacillus belonging to *Proteobacteria*, was associated with the decreased occurrence of adverse cardiovascular events during a three-year follow-up (HR: 0.545, 95%-CI: 0.387-0.773). The results of numerous clinically cohort and epidemiologic studies were also consistent with previous finding [13, 21]. The relationship of inflammation, dyslipidemia, and atherosclerosis have been well acknowledged [22]. In detail, increased levels of serum inflammatory cytokines [23], antibodies to periodontal pathogens [24, 25], and dyslipoproteins [26] have been observed in patients with atherosclerosis and periodontitis compared with patients keeping oral health. The higher OR for patients undergoing periodontal infection indicated that the occurrence of atherosclerotic cerebrovascular events was higher in patients with periodontitis [7, 13]. These results discussed above showed that periodontal infection might predispose to atherosclerotic cerebrovascular events.

Meanwhile, animal models infected with periodontal pathogen have been well conducted to confirm the role of this infection on the pathogenesis of cerebrovascular atherosclerosis. In the animal model, both ligature-induced periodontitis and lipopolysaccharide- (LPS-) induced periodontitis mimic human periodontitis in many aspects; they can cause alveolar bone loss and many phenotypes with a short time frame [27]. Putative or high-risk bacteria of periodontal diseases included *Aggregatibacter actinomycetemcomitans*, *Porphyromonas gingivalis*, *Tannerella forsythia*, *Treponema denticola*, and *Fusobacterium nucleatum* [5]. Zhang et al. [28] treated the apolipoprotein E-deficient spontaneously hyperlipidemic (apoE^-shl^) mice intravenously with *A. actinomycetemcomitans.* They found that the areas of atherosclerotic lesions and the serum levels of proinflammatory cytokines, chemokine, adhesion molecules, and proatherogenic factors (TLR2/4) were significantly elevated in experimental mice. These results suggested that systematic inflammatory reaction induced by periodontopathic bacteria might promote atherosclerotic events in apoE^-shl^ mice, and TLR2/4 might play a proatherogenic role on the aggravation of atherosclerosis. A similar phenomenon was observed in hyperlipidemic apoE^null^ mice infected with multiple or single periodontopathic bacteria (i.e., *T. forsythia* and *P. gingivalis*) [29–32].Meanwile, in an vitro trial, Triantafilou et al. [33] also observed that *P. gingivalis* LPS stimulated the proinflammatory cytokine production of human vascular endothelial via TLR2. On the contrary, Chukkapalli et al. [34] observed that a marked reduction of aortic plaque area and intimal thickness in TLR2^−/−^TLR4^−/−^-, TLR2^+/−^TLR4^−/−^-, and TLR2^−/−^TLR4^+/−^-deficient mice infected with multiple periodontal pathogens compared with normal group. Inaba et al. [35] found that the p38 and ERK1/2 phosphorylation, a potent activator of mitogen-activated protein kinase (MAPK) pathways acted as initiating inflammation, was decreased in TLR2 and TLR4 gene knockdown gingival epithelial cells. These results clearly demonstrated that partial or complete knockdown of gene of TLR2 and TLR4 reduces the progress of atherosclerotic plaque in infected animal models.

Lipoprotein oxidation is generally believed to be a key event in the initial stage of atherosclerosis [36]. In human, observational studies showed that the patient suffering from chronic periodontitis had higher levels of triglyceride (TG), very low-density lipoprotein (VLDL) as well as lipoprotein-associated phospholipase A2 (Lp-PLA2) which were treated as traditional atherosclerotic risk factors, and lower levels of HDL [37]. Liu et al. [38] explored the molecular mechanisms further; they found that *P. gingivalis-*LPS disturb cholesterol metabolism homeostasis via upregulation of acetyl CoA acyltransferase 1 (ACAT1) and downregulation of CD36 and ATP-binding cassette G1 (ABCG1), resulting in producing excess cholesterol ester and decreasing cellular cholesterol efflux. In addition, *P. gingivalis* trigger oxidation of high-density lipoprotein (HDL), which played an atheroprotective role on atherosclerosis by reversing cholesterol transport pathway and removing cholesterol from macrophages [39]. *P. gingivalis*-lysine-specific (Kgp) and arginine-specific (Rgp) gingipains, acting as proteolytic enzymes, were responsible for lipid peroxidation and antioxidants consume, contributing to increase oxidative stress [40]. These results suggested that periodontal microbiota induce lipoprotein dysfunction in atherosclerosis pathogenesis.

Atherosclerosis is a T-helper- (T_H_-) mediated disease [41]. In *P. gingivalis*-infected ApoE^−/−^mice, Yang et al. [42] found that the percentages of CD4^+^ T_H_17 cells were elevated along with the increased levels of T_h_17-related factors (i.e., ROR*γ*t, IL17) and decreased levels of T_reg_-related cytokines (i.e., FoxP3, IL-10, and TGF*β*1). *P. gingivalis* might disrupt the balance of T_h_17 and T_reg_ and finally lead to the imbalance of atheroprotective and proatherogenic cytokines. Moreover, Cai et al. [43] also observed that the proportion of T_h_17 cells, the major subtype of CD4^+^T cells, was significantly increased in ApoE-deficient mice with the elevated mRNA expression of Th17-related genes (i.e., IL-6, TGF-*β*, ROR*γ*t, and STAT3). Those T cells released plenty of proinflammatory cytokines, which also activated macrophages, B cells, monocytes, and other components of the plaque. The presence of DNA from periodontal bacteria also was detected in other host immune cells such as dendritic cells and macrophages, which can explain how periodontal pathogens reach to atherosclerotic lesions distant from primary periodontium [44]. *P. gingivalis*, *T. denticola*, *B. forsythus*, *Streptococcus mutans*-GS 5, and *S. gordonii* were examined in the macrophage foam cell, and *P. gingivalis* LPS plays a potential role on inducing macrophages to modify native LDL [45].

In in vitro trial performed by Jotwani et al. [46], the result demonstrated that *P. gingivalis* fimbriae uptake by monocyte-derived dendritic cells (MDDS) can not only induce CD4^+^T cells proliferation but also stimulate followed-related Th1 responses. These findings suggested that T_h_ cells and associated cytokines contribute to immune response in the pathogenesis of atherosclerosis. In a review [14], heat-shock proteins originated from periodontopathic microbiota have been proposed to play a role on activating endothelial cells, promoting monocyte adhesion and migration, elevating foam cell formation and production of proinflammatory cytokines via TLRs, and stimulating platelet activation. Toxins of periodontal pathogens, such as proteases, adhesions, and lectins, have also been shown to impair the immune response of the host, by degradation of interleukins and enable atherosclerotic plaque formation by increasing proliferation of vascular smooth muscle cells and platelet aggregation [31, 47, 48].

Periodontitis can exacerbate the formation and progress of atherosclerotic accidents; periodontopathic microbiota might play a crucial role. Based on the concept of “microbiota-gut-brain axis,” scientists put forward the concept of “microbiota-oral-brain axis”; however, the latter has not yet been well recognized [49]. The successful establishment of animal models challenged with periodontopathic bacteria contributed to a better understanding of the pathogenic mechanisms of periodontopathic microbiota on atherosclerosis.

## 3. Periodontal Microbial Signature of Atherosclerotic Plaques

The alteration of periodontal microbiome composition has been previously reported in atherosclerotic vascular disease (ASVD). Much of the recent studies focused on discovering the difference in microbiome composition in oral, coronary, and carotid plaques from patients with and without periodontal infection. A comparative analysis performed by Kannosh et al. [50] showed that *Tannerella forsythensis* (T.f) and *Treponema denticola* (T.d) were the most and the least common bacteria causing atherosclerotic plaques (APs), respectively. The dominated periopathogenic bacteria of APs were dominated by *T. forsythensis* (53%), *P. gingivalis* (38%), *P. intermedia* (1%), *A. actinomycetemcomitans* (14%), and *T. denticola* (6%). Similarly, Isoshima et al. [51] used pyrosequence of 16S rRNA pyrosequencing to show that the majority families of the periodontal bacteria were *Burkholderiales*, *Bacillale*, and *Rhizobiales*, forming the soil bacterial family. Utilizing the techniques of microbial whole-genome sequencing, Brun et al. [52] and Koren et al. [53] showed that the most dominantly detected phyla of periopathogens in APs with periodontitis were *Proteobacteria*, *Actinobacteria*, *Firmicutes*, and *Bacteroidetes*. Current studies aiming to detect a variety of periodontal microorganisms in atherosclerotic and subgingival plaques obtained different results due to the differences in study cohorts selected, DNA detection techniques, and periodontal sample collection. The results of studies about altered periodontal microbiota in atherosclerosis are summarized in Table 1.

The adverse periodontopathogens reported in patients with atherosclerosis included *Porphyromonas gingivalis* (*P. g*) [54–57], *Prevotella nigrescens* (*P. n*) [54, 58], *Aggregatibacter actinomycetemcomitans* (*A. a*) [58–60], *Tannerella forsythia* (*T. f*) [60–62], *Treponema denticola* (*T. d*) [59, 60, 62], *Prevotella intermedia* (*P. i*) [58, 60]. Changes in periodotopathogenic bacterial composition of altherosclerotic plaques were also observed in mouse models with ligature-induced periodontitis or LPS-induced periodontitis. In hyperlipidemic apoE^null^ mice, the presence of *P.g*, *T.d*, and *T.f* genomic DNA detected via polymerase chain reaction with bacterium-specific primers was examined in multiple organ tissues, such as the aorta, heart, liver, and spleen [29]. Similarly, *T. forsythia* and its metabolite BspA promoted the foam cell formation and increased atherosclerotic lesion progression in ApoE^−/−^ mice with the elevated levels of lipid metabolism-related genes and declined expression of antiatherogenic genes [31]. The proatherogenic roles of *A. actinomycetemcomitans* [28] and *P.gingivalis* [39] were also proven in similar animal trials.

In summary, pathological evidence demonstrated that the high-risk proatherosclerotic microbiota are mainly followed by *Firmicutes*, *Actinobacteria*, *Bacteroidetes*, *Proteobacteria*, *Fusobacteria*, and *Spirochaetes*. Periodonpathic microbiota might enter into our blood circulation through our dairy activities (tooth brushing, chewing, and flossing) and reside in the wall of distant atherosclerotic vessels, leading to inflammation activation, immune mediation, dyslipidemia, oxidative stress, and endothelial dysfunction. However, the exact mechanism is still under exploration.

## 4. Role of TLR Signaling in Periodonpathic Pathogen-Induced Atherosclerosis

Toll-like receptors (TLRs), the most characteristic members of pattern recognition receptors (PRRs), served as recognizing pathogens and its metabolites and activating the immune and inflammatory response [63]. The potential mechanisms of periodonpathic pathogen-induced atherosclerosis currently under discussion include inflammation, immunity, lipoprotein oxidation, and endothelial dysfunction [14, 64, 65]. This study mainly focused on discussing inflammatory mechanisms. Specifically, periodontal microbiota and its metabolites were presumed to play a role in triggering a series of inflammatory responses via the TLR–NF-*κ*B signaling pathway.

### 4.1. Toll-Like-Receptor Acted as the Trigger and Recognizer of Inflammation Reaction

TLRs, the most characteristic members of pattern recognition receptors, are widely expressed in the various cell membrane or located in the cytoplasm, and the elevated levels of TLR expression in macrophages and endothelial cells of atheroma have been observed [66]. Inaba et al. [35] demonstrated that *P. gingivalis* LPS enhanced the expression of TLR2 and TLR4 mRNA in human gingivalis epithelial cells. In vivo trial performed by Nakamura et al. [67] also suggested that TLR2 might take charge for recognizing the fraction of *P. gingivalis* LPS and mediating mononuclear cell adhesion to the vascular endothelium. TLR2 blockade or deficiency inhibited the process of CD11b/CD18-dependent adhesion of human monocytes and transmigration in *P. gingivalis*-induced mouse models [68]. Chukkapalli et al. [69] also observed that the levels of local and systematic inflammation were not increased in polymicrobial-infected TLR2/4^−/−^ mice with decreased accelerated alveolar bone resorption (ABR) and intrabony defects. Further, Huang et al. [70] found that the GroEL protein from *P. gingivalis*, belonging to the heat shock protein (HSP) 60 family, upregulated the expression of TLR4 in human coronary artery endothelial cells (HCAECs) in vitro and on aortas of high-cholesterol-fed B57BL/6 and B57BL/6Tlr4^lps-del^ mice in vivo and matched with TLR4. Uehara et al. [71] demonstrated that gingipains (HRgpA, RgpB, and Kgp) originating from *P. gingivalis* and Toll-like receptor played a synergistic role on triggering the production of proinflammatory cytokines from THP-1 cells and mediating monocyte migration. Li et al. [17] summarized the role of pathogen-mediated atherosclerosis and pointed out that TLR2 and TLR4 had a significant impact on periodontal pathogen-induced atherosclerotic disease. These results suggested that the pathological effects of periodontopathogenic bacteria were mainly due to their ability to release a series of virulence factors, such as HSP, gingipains, and LPS through TLRs (mainly TLR2/4).

It is well acknowledged that the primary trigger of inflammatory response in atherosclerosis is driven by lipid modification [36, 72], chronic infection [73], low-grade endotoxemia, and inflammatory cascade in which the circulating cytokines bind to the signaling receptors and ligands and create a vicious circle [74, 75]. Lin et al. [76] observed that the expression of serum inflammatory cytokines (i.e., CRP, IL-6, TNF-*α*, and MCP-1) and TLR2-TLR4 of local aortic tissues significantly increased in the rabbits with intravenous injection of *P. gingivalis*-LPS. In agreement with several in vivo studies [77–80], the ligature or LPS-induced periodontitis promoted the expression of systemic proinflammatory markers and adhesion molecules, including TNF-*α*, IL-1*β*, IL-6, CRP, TGF-1*β*, IFN-*γ*, ICAM-1, VCAM-1, and PECAM-1, which in turn exacerbated the process of atherosclerosis in the ApoE^−/−^ mice. Meanwhile, these inflammatory factors aggravated the endothelial dysfunction, promoted LDL oxidation, induced diversified immune cell migration, and exacerbated macrophage foam cell formation, resulting in the atherosclerosis of the vessel wall. The plasma levels of LDL and its core protein ApoB and other antigens cause CD4+ T-helper cell activation through TLRs and differentiation into functionally distinct T-helper subtype cells, which further release both atheroprotective or proatherogenic cytokines (i.e., TGF-*β*, IFN-*γ*, TNF-*α*, IL-4, IL-5, IL-10, IL-13, IL-17, IL-21, and IL-23) [41]. Interestingly, the initial atheroprotective cytokines may function as proatherogenic cytokines at different stages of atherosclerosis. Specifically, IL-10 and TGF-*β* play an atheroprotective role. Also, IFN-*γ* and TNF-*α* play a proatherogenic role, which the roles of IL-4, IL-5, IL-13, IL-17, IL-21, and IL-23 are still in exploration. Collectively, TLR recognized the pathogen-associated antigens (i.e., LPS, and ox-LDL), activated innate/adaptive immune cells, and released various cytokines (Table 2).

### 4.2. Role of TLR-NF-*κ*B-Signaling in Periodontopathogenic Bacteria-Mediated Atherosclerosis

As discussed earlier, TLR (mainly TLR2 and TLR4) played a crucial role in recognizing periodontal microbiota and its metabolites. TLR family members and every subtype recognize specific ligands including LPS, and nucleic acid, or a restricted repertoire of ligands such as lipids or certain proteins. In addition, each TLR has diversified signal adaptor molecules (MyD88, TRAM, TIRAP, and TRIF/TICAM1), and they are engaged by delivering inflammatory signaling and thus initiating the fight against pathogens [63]. TIRAP and MyD88 recruited by TLR2/4 are responsible for activating NF-*κ*B and MAPK, and NF-*κ*B is transferred to the nucleus and serves as the transcription factor for the genes encoding proinflammatory chemokines and cytokines [16]. The activation of NF-*κ*B and MAPK becomes the critical point in various signaling pathways initiated by TLRs which influence the subsequent expression of numerous immune and inflammation-related genes [63].

Lin et al. [76] observed a significant increase in the levels of inflammatory cytokines accompanied by the increased expression of TLR2/4 and several signaling molecules including NF-𝜅B p38 mitogen-activated protein kinase (p38-MAPK), and Jun N-terminal kinase (JNK) in rabbits injected with *P. gingivalis*. Their results suggested that NF-𝜅B and MAPK signaling pathways were responsible for upregulating inflammatory reactions, and the antagonists of these pathways might be effective against atherosclerosis. Also, endothelial dysfunction is the first step of atherosclerosis. Xie et al. [81] demonstrated that using the antagonist or inhibitor of the TLR- NF-𝜅B pathway could rescue the damage of endothelial homeostasis damaged by *P. gingivalis*. Also, they performed another experiment suggesting that the downregulation of BMAL1, a core clock gene, could influence the functions of the TLRs-NF-*κ*B signaling axis and exacerbate *P. gingivalis*-induced atherosclerosis via promoting oxidative stress [82]. Therefore, Xie et al. proposed a rational hypothesis that BMAL1 acted as an intermediary signaling molecule, and the NF-*κ*B-BMAL1 axis amplified oxidative stress of the endothelium, contributing to subsequent monocyte adhesion, proinflammatory cytokine release, and endothelial cell apoptosis. An excellent in vitro validation of the mechanism has been provided by Li et al. [83] They demonstrated that *P. gingivalis*, one of the main periodontal pathogens, participated in the process of monocyte migration and adhesion to human umbilical vein endothelial cells (HUVECs) via oxidized low-density lipoprotein receptor-1 (LOX-1). They also showed that the activation of LOX-1 on endothelial cells increased the expression of monocyte chemoattractant protein-1 (MCP-1), intercellular adhesion molecule-1 (ICAM-1), E-selectin, and other adhesion molecules. MCP-1 bound to its ligand C–C chemokine receptor (CCR2)/Integrin *α*M*β*2 on THP-1 cells prompting monocyte migration. They proved the hypothesis that the mutual interaction of *P. gingivalis*, NF-*κ*B, and LOX-1 provided a positive feedback loop and functioned as an “inflammation amplifier” or “local cytokine storm,” leading to a cascade of downstream inflammatory responses.

**Figure 2:**
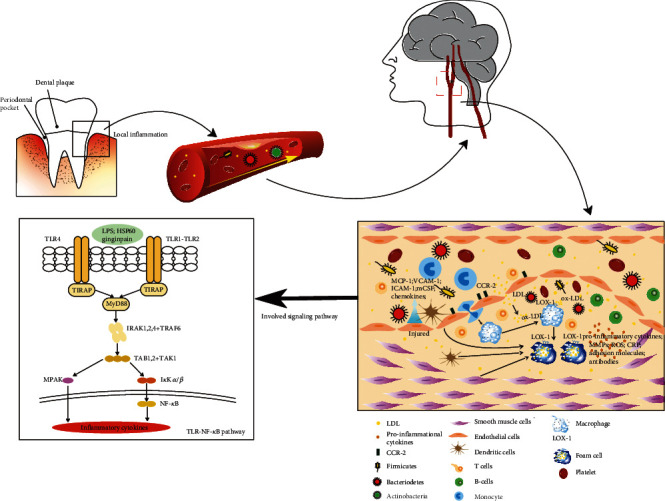
Schematic representation of inflammatory mechanisms linking periopathogens to the development of atherosclerosis. Periodontal dysbacteriosis induces local inflammation, and etiopathogenic bacteria that resided in the subgingival pocket can enter into the bloodstream. Next, TLR binds to pathogens and transduces a downstream signaling molecule and result in the activation of various cells and the release of proinflammatory cytokines, which work together to promote the formation of atherosclerotic plaques.

Based on the aforementioned discussion, the TLR2/4-NF-*κ*B/MAPK signaling pathway seems to play a critical role in the formation and development of atherosclerosis. The specific mechanism is shown in Figure 2. Moreover, targeting these pathways might serve as a promising treatment in the future.

## 5. Effects of Targeted-Periodontopathogen Treatment on Atherosclerosis

Considering the role of periodontopathic microbiota in atherosclerosis, controlling and eliminating the etiologic factors are of significance. However, the effects of treatment involving targeted periodontal microbiota is still not clearly understood. A population-based study (*n* = 247, 696) in Korea performed by Park et al. showed that regular tooth brushing and professional cleaning were shown to decrease the prevalence of atherosclerotic diseases by 9% and 14%, respectively [84]. Their results suggested that maintaining oral hygiene might reduce the risk of cardiovascular and cerebrovascular events, which was in accordance with several observational cohort studies conducted by Lin et al. [85], Kudo et al. [86], and Chen et al. [87] Periodontopathic microbiota existed on the dental surface. The periodontal pocket was the main pathogen of periodontal diseases, contributing to local and systematic inflammation. Regular tooth brushing and professional cleaning are the effective ways to reduce the accumulation of bacterial plaques on teeth.

Current studies on anti-infectious treatment are limited. In animal models, treatment with antibiotics (i.e., metronidazole and amoxicillin) could only temporarily relieve periodontitis, decrease the levels of inflammatory cytokines and metalloproteases, and reduce atherosclerotic plaque burden. Xie et al. [82] reported that the Oil Red O staining of atherosclerotic lesions in the aorta and aortic root has reduced, and the expression of IL-1*β*, IL6, and TNF-*α* in serum had significantly decreased in the model of *P. gingivalis*-infected ApoE-deficient mice treated with metronidazole (MTZ) or/and melatonin (MLT). Interestingly, they also found that the expression of BMAL-1, which has been known as the gene regulating circadian clock, was restored to the normal level, and the elevated production of superoxide radical and proinflammatory cytokines was efficiently reversed. Rajendran et al. [88] showed that the treatment with antibiotics (AB) influenced the expression of gingival tissue inflammation markers and inhibited the growth of inflammatory blood myeloid dendritic cells and T_reg_–Th17 plasticity in 4-6 weeks after antibiotics. However, the limitation of this study was the short duration of periodontitis induction and antibiotics treatment in comparison to periodontitis and atherosclerosis both chronic inflammatory diseases in humans. Nevertheless, it would require a large randomized controlled trial to demonstrate whether the antibiotic therapy of periodontitis can affect atherosclerotic cardiovascular diseases. Xiu et al. [89] examined the gum microbiota and found that the combined periodontal treatments, tooth extraction, and anti-inflammatory therapy significantly reduced the absolute numbers of periodontal pathogens and decreased the level of bacteremia in mice. Aspirin and statin are both commonly used as secondary preventive medicine in cerebrovascular diseases. The anti-inflammatory and antibiotic treatments had decreased the level of circulating cytokines. Hence, it is worth exploring whether there is prevention or reduction is possible in cardiovascular events. Meanwhile, long-term antibiotic therapy could inhibit or kill the normal dominant flora of human body and lead flora disorder, it is also worth pondering the length of antibiotic therapy. This may require larger clinical trials in the future.

The therapies aimed at proinflammatory cytokines and TLR–NF-*κ*B signaling pathway are other novel ways to attenuate atherosclerosis. In the mouse models, Rekhi et al. found that the decreased levels of proinflammatory cytokines and reduction of atherosclerotic plaques in the aorta were observed in the male LDLR KO mice treated with AP5055, an inhibitor of CD36 that inhibited NF-*κ*B activity [90]. Pan et al. also proved that rosiglitazone, the PPAR*γ* agonist, acted as an insulin-sensitizing agent in the clinical management of type 2 diabetes mellitus. It can attenuate *P. gingivalis*-induced atherosclerosis by decreasing the expression of inflammatory cytokines and TLR2/4, downregulating NF-*κ*B activity, and lowering the levels of serum lipoproteins [91]. Chukkapalli et al. also observed that the progression of atherosclerosis significantly slowed down in polymicrobiota-infected TLR2^−/−^ and TLR4^−/−^ mice [69]. These findings suggested that disturbing the TLR/nuclear factor-*κ*B pathway could attenuate periodontopathogen-induced atherosclerosis and decrease the level of systematic inflammation. However, clinical evidence from humans to support the therapy targeting proinflammatory cytokines is insufficient. In the 2018 CANTOS trial, patients treated with canakinumab, a monoclonal antibody targeting interleukin- (IL-) 1*β* via IL-6 signaling pathway, showed a decrease in the levels of IL-6 with a 32% reduction in detrimental atherosclerotic events and a 52% reduction in cardiovascular mortality compared with control individuals [92]. These results suggested that the potential benefits of canakinumab existed in atherosclerotic diseases. Nevertheless, canakinumab is still not widely available for clinical treatment due to the high expenses involved in the whole therapy and limited human trials.

## 6. Conclusion and Outlook

The multiple microbiome-related and immunochemistry studies supported that pathogenic periodontal microbiota was involved in the pathogenesis of atherosclerosis. A potential role for TLR 2/4-NF-*κ*B signaling pathway in the link between periodontopathic microbiota and the progress of atherosclerosis was preliminarily verified, including recognizing exogenous antigens, transmitting inflammatory signaling, and promoting the release of cytokines and cells. The improvement in oral hygiene is effective in reducing atherosclerotic risks. However, current therapeutic measures are limited, and large cohort studies to verify the conclusion from animal trials are lacking. Plausible explanations may be as follows: (1) the side effects of long-term anti-infection treatment for normal flora; (2) the high expenses of anti-inflammatory agents, and (3) the damage to vessel walls during effective therapy. Hence, the main problems to address are elucidating the detailed role of periodontopathogenic microbiota invasion of atheromas, except the influence of bacteremia from other sites such as gut flora and respiratory flora, and effective therapy to inflammatory response between periodontal infection and atherosclerosis. In summary, pathogenic periodontal microbiota has been suggested to exert a critical role in the initiation and progress of atherosclerosis via the TLR 2/4–NF-*κ*B signaling pathway. Further studies should focus on how to take measures to target this signaling so as to prevent and treat periodontal infection-induced atherosclerosis.

## Figures and Tables

**Table 1:** The main phylum difference of oral bacteria in the periodontal and atherosclerotic plaque.

Phylum	Koren et al. [53]		Isoshima et al. [51]	Ismail et al. [93]		Pavlic et al. [60]		Brun et al. [52]		Kannosh et al. [50]		Fak et al. [94]		
	Oral	APs	Oral	APs	Oral	APs	Oral	APs	Oral	APs	Oral	APs	Oral	APs
Firmicutes	+	↓		↑	↑	—			—	↑			—	↑
Bacteroidetes	+	+			↑	—	+	+	—	↑	+	+	—	↑
Actinobacteria	+	+	↓			—	+	+	—	↑	+	+	—	↑
Fusobacteria	+		↑		↑	—	+	+	—	+	↑	↑	—	↑
Proteobacteria	+	↑	↓	↑		—			—	↑			—	↑
Spirochaetes	+					—	+	+	—	+	+	+	—	
Acidobacteria		+		↑		—			—				—	
Aquificae			↑			—			—				—	
Other (<1%)	+					—			—				—	

^∗^APs: atherosclerotic plaques.

**Table 2:** Related cells and cytokines involved in atherosclerosis.

Cells	Cytokines	Others
Immune cells: DCs, macrophages, monocyte, T cells (Treg, TR1, TH17, TFH, TH2, TH1), B cells (Be1/2, Breg, IRA-B), NK cells	ILs family: IL-1*β*, IL-4, IL-5, IL-6, IL-10, IL-12, IL-13,IL-17, IL-18, IL-21, IL-23 Chemokine: CCL-2, CCL-3, CCL-5, CXCL-8 Growth factor: VEGF, PDGF, IFN-*γ*, TNF-*α*, GM-CSF, TGF-*β*	Adhesion molecules: VCAM-1, ICAM-1, PECAM-1 MMPs: MMP-8, MMP-9, CRP, NO, ROS, SAA, haptoglobin, fibrinogen
Nonimmune cells: ECs, VSMCs, EPIs, fibroblast		

## Data Availability

No data were used to support this study.

## References

[1] Kumar S. (2019). Evidence-based update on diagnosis and management of gingivitis and periodontitis. *Dental Clinics of North America*.

[2] Eke P. I., Borgnakke W. S., Genco R. J. (2000). Recent epidemiologic trends in periodontitis in the USA. *Periodontology*.

[3] Bui F. Q., Almeida-da-Silva C. L. C., Huynh B. (2019). Association between periodontal pathogens and systemic disease. *Biomedical Journal*.

[4] Geovanini G. R., Libby P. (2018). Atherosclerosis and inflammation: overview and updates. *Clinical Science (London, England)*.

[5] Bale B. F., Doneen A. L., Vigerust D. J. (2017). High-risk periodontal pathogens contribute to the pathogenesis of atherosclerosis. *Postgraduate Medical Journal*.

[6] Schulz S., Schlitt A., Hofmann B., Schaller H. G., Reichert S. (2020). Periodontal pathogens and their role in cardiovascular outcome. *Journal of Clinical Periodontology*.

[7] Pradeep A. R., Hadge P., Arjun Raju P., Shetty S. R., Shareef K., Guruprasad C. N. (2010). Periodontitis as a risk factor for cerebrovascular accident: a case-control study in the Indian population. *Journal of Periodontal Research*.

[8] Aoyama N., Suzuki J. I., Kobayashi N. (2017). Periodontitis deteriorates peripheral arterial disease in Japanese population via enhanced systemic inflammation. *Heart and Vessels*.

[9] Benjamin E. J., Virani S. S., Callaway C. W. (2018). Heart disease and stroke statistics-2018 update: a report from the American Heart Association. *Circulation*.

[10] Mozos I., Stoian D., El-Esawi M. A. (2022). Oral health and cardiovascular disorders. *Understanding the Molecular Crosstalk in Biological Processes*.

[11] Desvarieux M., Demmer R. T., Rundek T. (2003). Relationship between periodontal disease, tooth loss, and carotid artery plaque: the oral infections and vascular disease epidemiology study (INVEST). *Stroke*.

[12] Tiensripojamarn N., Lertpimonchai A., Tavedhikul K. (2021). Periodontitis is associated with cardiovascular diseases: a 13-year study. *Journal of Clinical Periodontology*.

[13] Patel U. K., Malik P., Kodumuri N. (2020). Chronic periodontitis is associated with cerebral atherosclerosis -a nationwide study. *Cureus*.

[14] Schenkein H. A., Papapanou P. N., Genco R., Sanz M. (2020). Mechanisms underlying the association between periodontitis and atherosclerotic disease. *Periodontology*.

[15] Friedewald V. E., Kornman K. S., Beck J. D. (2009). The American Journal of Cardiology and Journal of Periodontology Editors' Consensus: periodontitis and atherosclerotic cardiovascular disease. *The American Journal of Cardiology*.

[16] Sharma S., Garg I., Ashraf M. Z. (2016). TLR signalling and association of TLR polymorphism with cardiovascular diseases. *Vascular Pharmacology*.

[17] Li B., Xia Y., Hu B. (2020). Infection and atherosclerosis: TLR-dependent pathways. *Cellular and Molecular Life Sciences*.

[18] Ross R. (1999). Atherosclerosis--an inflammatory disease. *The New England Journal of Medicine*.

[19] Wu T., Trevisan M., Genco R. J., Dorn J. P., Falkner K. L., Sempos C. T. (2000). Periodontal disease and risk of cerebrovascular disease: the first national health and nutrition examination survey and its follow-up study. *Archives of Internal Medicine*.

[20] Boillot A., Demmer R. T., Mallat Z. (2015). Periodontal microbiota and phospholipases: the oral infections and vascular disease epidemiology study (INVEST). *Atherosclerosis*.

[21] Zeng X. T., Leng W. D., Lam Y. Y. (2016). Periodontal disease and carotid atherosclerosis: a meta-analysis of 17, 330 participants. *International Journal of Cardiology*.

[22] Libby P., Ridker P. M., Maseri A. (2002). Inflammation and atherosclerosis. *Circulation*.

[23] Wytrykowska A., Prosba-Mackiewicz M., Nyka W. M. (2016). IL-1*β*, TNF-*α*, and IL-6 levels in gingival fluid and serum of patients with ischemic stroke. *Journal of Oral Science*.

[24] Pussinen P. J., Alfthan G., Rissanen H., Reunanen A., Asikainen S., Knekt P. (2004). Antibodies to periodontal pathogens and stroke risk. *Stroke*.

[25] Hosomi N., Aoki S., Matsuo K. (2012). Association of serum anti-periodontal pathogen antibody with ischemic stroke. *Cerebrovascular Diseases*.

[26] Chen C., Zhu J., Deng X. (2021). Severe periodontitis is associated with the serum levels of hypersensitive C reactive protein and lipoprotein-associated phospholipase A2 in the patients of acute ischemic stroke. *Journal of Clinical Neuroscience*.

[27] Abe T., Hajishengallis G. (2013). Optimization of the ligature-induced periodontitis model in mice. *Journal of Immunological Methods*.

[28] Zhang T., Kurita-Ochiai T., Hashizume T., du Y., Oguchi S., Yamamoto M. (2010). Aggregatibacter actinomycetemcomitans accelerates atherosclerosis with an increase in atherogenic factors in spontaneously hyperlipidemic mice. *FEMS Immunology and Medical Microbiology*.

[29] Rivera M. F., Lee J. Y., Aneja M. (2013). Polymicrobial infection with major periodontal pathogens induced periodontal disease and aortic atherosclerosis in hyperlipidemic ApoE (null) mice. *PLoS One*.

[30] Chukkapalli S. S., Velsko I. M., Rivera-Kweh M. F., Zheng D., Lucas A. R., Kesavalu L. (2015). Polymicrobial oral infection with four periodontal bacteria orchestrates a distinct inflammatory response and atherosclerosis in ApoE null mice. *PLoS One*.

[31] Lee H. R., Jun H. K., Choi B. K. (2014). Tannerella forsythia BspA increases the risk factors for atherosclerosis in ApoE−/− mice. *Oral Diseases*.

[32] Chukkapalli S. S., Rivera-Kweh M. F., Velsko I. M. (2015). Chronic oral infection with major periodontal bacteria Tannerella forsythia modulates systemic atherosclerosis risk factors and inflammatory markers. *Pathogens and Disease*.

[33] Triantafilou M., Gamper F. G., Lepper P. M. (2007). Lipopolysaccharides from atherosclerosis-associated bacteria antagonize TLR4, induce formation of TLR2/1/CD36 complexes in lipid rafts and trigger TLR2-induced inflammatory responses in human vascular endothelial cells. *Cellular Microbiology*.

[34] Chukkapalli S. S., Ambadapadi S., Varkoly K. (2018). Impaired innate immune signaling due to combined Toll-like receptor 2 and 4 deficiency affects both periodontitis and atherosclerosis in response to polybacterial infection. *Pathogens and Disease*.

[35] Inaba H., Yoshida S., Nomura R. (2020). Porphyromonas gulae lipopolysaccharide elicits inflammatory responses through toll-like receptor 2 and 4 in human gingivalis epithelial cells. *Cellular Microbiology*.

[36] van der Valk F. M., Bekkering S., Kroon J. (2016). Oxidized phospholipids on lipoprotein (a) elicit arterial wall inflammation and an inflammatory monocyte response in humans. *Circulation*.

[37] Koshy B. S., Mahendra J. (2017). The association between periodontal status, serum lipid levels, lipoprotein associated phosholipase A2 (Lp-PLA2) in chronic periodontitis subjects and healthy controls. *Journal of Clinical and Diagnostic Research*.

[38] Liu F., Wang Y., Xu J., Liu F., Hu R., Deng H. (2016). Effects of <i>Porphyromonas gingivalis</i> lipopolysaccharide on the expression of key genes involved in cholesterol metabolism in macrophages. *Archives of Medical Science*.

[39] Kim H. J., Cha G. S., Kim H. J. (2018). Porphyromonas gingivalis accelerates atherosclerosis through oxidation of high-density lipoprotein. *Journal of Periodontal & Implant Science*.

[40] Lönn J., Ljunggren S., Klarström-Engström K., Demirel I., Bengtsson T., Karlsson H. (2018). Lipoprotein modifications by gingipains of Porphyromonas gingivalis. *Journal of Periodontal Research*.

[41] Wolf D., Ley K. (2019). Immunity and inflammation in atherosclerosis. *Circulation Research*.

[42] Yang J., Wu J., Zhang R. (2017). *Porphyromonas gingivalis* oral infection promote T helper 17/Treg imbalance in the development of atherosclerosis. *Journal of Dental Sciences*.

[43] Cai Y., Kobayashi R., Hashizume-Takizawa T., Kurita-Ochiai T. (2014). _Porphyromonas gingivalis_ infection enhances Th17 responses for development of atherosclerosis. *Archives of Oral Biology*.

[44] Slocum C., Kramer C., Genco C. A. (2016). Immune dysregulation mediated by the oral microbiome: potential link to chronic inflammation and atherosclerosis. *Journal of Internal Medicine*.

[45] Qi M., Miyakawa H., Kuramitsu H. K. (2003). Porphyromonas gingivalis induces murine macrophage foam cell formation. *Microbial Pathogenesis*.

[46] Jotwani R., Cutler C. W. (2004). Fimbriated Porphyromonas gingivalis is more efficient than fimbria-deficient P. gingivalis in entering human dendritic cells in vitro and induces an inflammatory Th1 effector response. *Infection and Immunity*.

[47] Komatsu T., Nagano K., Sugiura S. (2012). E-selectin mediates Porphyromonas gingivalis adherence to human endothelial cells. *Infection and Immunity*.

[48] Wan M., Liu J. R., Wu D., Chi X. P., Ouyang X. Y. (2015). E-selectin expression induced by Porphyromonas gingivalis in human endothelial cells via nucleotide-binding oligomerization domain-like receptors and Toll-like receptors. *Molecular Oral Microbiology*.

[49] Tonomura S., Ihara M., Friedland R. P. (2020). Microbiota in cerebrovascular disease: a key player and future therapeutic target. *Journal of Cerebral Blood Flow & Metabolism*.

[50] Kannosh I., Staletovic D., Toljic B. (2018). The presence of periopathogenic bacteria in subgingival and atherosclerotic plaques - an age related comparative analysis. *Journal of Infection in Developing Countries*.

[51] Isoshima D., Yamashiro K., Matsunaga K. (2021). Microbiome composition comparison in oral and atherosclerotic plaque from patients with and without periodontitis. *Odontology*.

[52] Brun A., Nuzzo A., Prouvost B. (2021). Oral microbiota and atherothrombotic carotid plaque vulnerability in periodontitis patients. A cross-sectional study. *Journal of Periodontal Research*.

[53] Koren O., Spor A., Felin J. (2011). Human oral, gut, and plaque microbiota in patients with atherosclerosis. *Proceedings of the National Academy of Sciences of the United States of America*.

[54] Yakob M., Söder B., Meurman J. H., Jogestrand T., Nowak J., Söder P. Ö. (2011). Prevotella nigrescens and Porphyromonas gingivalis are associated with signs of carotid atherosclerosis in subjects with and without periodontitis. *Journal of Periodontal Research*.

[55] Pussinen P. J., Alfthan G., Jousilahti P., Paju S., Tuomilehto J. (2007). Systemic exposure to Porphyromonas gingivalis predicts incident stroke. *Atherosclerosis*.

[56] Kudo C., Shin W. S., Minabe M. (2015). Analysis of the relationship between periodontal disease and atherosclerosis within a local clinical system: a cross-sectional observational pilot study. *Odontology*.

[57] Chistiakov D. A., Orekhov A. N., Bobryshev Y. V. (2016). Links between atherosclerotic and periodontal disease. *Experimental and Molecular Pathology*.

[58] Gaetti-Jardim E., Marcelino S. L., Feitosa A. C., Romito G. A., Avila-Campos M. J. (2009). Quantitative detection of periodontopathic bacteria in atherosclerotic plaques from coronary arteries. *Journal of Medical Microbiology*.

[59] Aquino A. R. L., Lima K. C., Paiva M. S., Rôças I. N., Siqueira J. F. (2011). Molecular survey of atheromatous plaques for the presence of DNA from periodontal bacterial pathogens, archaea and fungi. *Journal of Periodontal Research*.

[60] Pavlic V., Peric D., Kalezic I. S. (2021). Identification of periopathogens in atheromatous plaques obtained from carotid and coronary arteries. *BioMed Research International*.

[61] Ardila C. M., Perez-Valencia A. Y., Rendon-Osorio W. L. (2015). Tannerella forsythia is associated with increased levels of atherogenic low density lipoprotein and total cholesterol in chronic periodontitis. *Journal of Clinical and Experimental Dentistry*.

[62] Mahalakshmi K., Krishnan P., Arumugam S. B. (2017). "Association of periodontopathic anaerobic bacterial co-occurrence to atherosclerosis" - a cross-sectional study. *Anaerobe*.

[63] Kawai T., Akira S. (2009). The roles of TLRs, RLRs and NLRs in pathogen recognition. *International Immunology*.

[64] Tuominen H., Taina M., Puranen M., Onatsu J., Huumonen S., Vanninen R. (2020). Serum high-sensitive C-reactive protein may reflect periodontitis in patients with stroke. *In Vivo*.

[65] Aarabi G., Heydecke G., Seedorf U. (2018). Roles of oral infections in the pathomechanism of atherosclerosis. *International Journal of Molecular Sciences*.

[66] Edfeldt K., Swedenborg J., Hansson G. K., Yan Z. Q. (2002). Expression of toll-like receptors in human atherosclerotic lesions: a possible pathway for plaque activation. *Circulation*.

[67] Nakamura N., Yoshida M., Umeda M. (2008). Extended exposure of lipopolysaccharide fraction from _Porphyromonas gingivalis_ facilitates mononuclear cell adhesion to vascular endothelium via Toll-like receptor-2 dependent mechanism. *Atherosclerosis*.

[68] Harokopakis E., Albzreh M. H., Martin M. H., Hajishengallis G. (2006). TLR2 transmodulates monocyte adhesion and transmigration via Rac 1- and PI3K-mediated inside-out signaling in response to Porphyromonas gingivalis fimbriae. *Journal of Immunology*.

[69] Chukkapalli S. S., Velsko I. M., Rivera‐Kweh M. F., Larjava H., Lucas A. R., Kesavalu L. (2017). Global TLR2 and 4 deficiency in mice impacts bone resorption, inflammatory markers and atherosclerosis to polymicrobial infection. *Molecular Oral Microbiology*.

[70] Huang C. Y., Shih C. M., Tsao N. W. (2016). The GroEL protein of Porphyromonas gingivalis regulates atherogenic phenomena in endothelial cells mediated by upregulating toll-like receptor 4 expression. *American Journal of Translational Research*.

[71] Uehara A., Imamura T., Potempa J., Travis J., Takada H. (2008). Gingipains from Porphyromonas gingivalis synergistically induce the production of proinflammatory cytokines through protease-activated receptors with Toll-like receptor and NOD1/2 ligands in human monocytic cells. *Cellular Microbiology*.

[72] Mauricio D., Castelblanco E., Alonso N. (2020). Cholesterol and inflammation in atherosclerosis: an immune-metabolic hypothesis. *Nutrients*.

[73] Shah P. K. (2019). Inflammation, infection and atherosclerosis. *Trends in Cardiovascular Medicine*.

[74] Hilgendorf I., Theurl I., Gerhardt L. M. (2014). Innate response activator B cells aggravate atherosclerosis by stimulating T helper-1 adaptive immunity. *Circulation*.

[75] Wolf D., Zirlik A., Ley K. (2015). Beyond vascular inflammation--recent advances in understanding atherosclerosis. *Cellular and Molecular Life Sciences*.

[76] Lin G., Chen S., Lei L. (2015). Effects of intravenous injection of *Porphyromonas gingivalis* on rabbit inflammatory immune response and atherosclerosis. *Mediators of Inflammation*.

[77] Campi P., Herrera B. S., de Jesus F. N. (2016). Endothelial dysfunction in rats with ligature-induced periodontitis: participation of nitric oxide and cycloxygenase-2-derived products. *Archives of Oral Biology*.

[78] Chi L., Cheng X., He X. (2019). Increased cortical infarction and neuroinflammation in ischemic stroke mice with experimental periodontitis. *Neuroreport*.

[79] O'Boyle C., Haley M. J., Lemarchand E. (2020). Ligature-induced periodontitis induces systemic inflammation but does not alter acute outcome after stroke in mice. *International Journal of Stroke*.

[80] Velsko I. M., Chukkapalli S. S., Rivera M. F. (2014). Active invasion of oral and aortic tissues by Porphyromonas gingivalis in mice causally links periodontitis and atherosclerosis. *PLoS One*.

[81] Xie M., Tang Q., Yu S. (2020). _Porphyromonas gingivalis_ disrupts vascular endothelial homeostasis in a TLR-NF- *κ*B axis dependent manner. *International Journal of Oral Science*.

[82] Xie M., Tang Q., Nie J. (2020). BMAL1-downregulation aggravates <i>Porphyromonas gingivalis</i>-induced atherosclerosis by encouraging oxidative stress. *Circulation Research*.

[83] Li Q., Liu J., Liu W. (2020). LOX-1 regulates P. gingivalis-induced monocyte migration and adhesion to human umbilical vein endothelial cells. *Frontiers in Cell and Development Biology*.

[84] Park S. Y., Kim S. H., Kang S. H. (2019). Improved oral hygiene care attenuates the cardiovascular risk of oral health disease: a population-based study from Korea. *European Heart Journal*.

[85] Lin H. W., Chen C. M., Yeh Y. C. (2019). Dental treatment procedures for periodontal disease and the subsequent risk of ischaemic stroke: a retrospective population-based cohort study. *Journal of Clinical Periodontology*.

[86] Kudo C., Shin W. S., Sasaki N. (2018). Effects of periodontal treatment on carotid intima-media thickness in patients with lifestyle-related diseases: Japanese prospective multicentre observational study. *Odontology*.

[87] Chen Z. Y., Chiang C. H., Huang C. C. (2012). The association of tooth scaling and decreased cardiovascular disease: a nationwide population-based study. *The American Journal of Medicine*.

[88] Rajendran M., Looney S., Singh N. (2019). Systemic antibiotic therapy reduces circulating inflammatory dendritic cells and Treg-Th17 plasticity in periodontitis. *Journal of Immunology*.

[89] Xiuyun R., Chong W., Xin L. (2017). Effects of oral interventions on carotid artery in rats with chronic periodontitis for the detection of Porphyromonas gingivalis and the expression of C-reactive protein. *Hua Xi Kou Qiang Yi Xue Za Zhi*.

[90] Rekhi U. R., Catunda R. Q., Alexiou M., Sharma M., Fong A., Febbraio M. (2021). Impact of a CD36 inhibitor on Porphyromonas gingivalis mediated atherosclerosis. *Archives of Oral Biology*.

[91] Pan S., Lei L., Chen S., Li H., Yan F. (2014). Rosiglitazone impedes Porphyromonas gingivalis-accelerated atherosclerosis by downregulating the TLR/NF-*κ*B signaling pathway in atherosclerotic mice. *International Immunopharmacology*.

[92] Ridker P. M., Libby P., MacFadyen J. G. (2018). Modulation of the interleukin-6 signalling pathway and incidence rates of atherosclerotic events and all-cause mortality: analyses from the Canakinumab Anti-Inflammatory Thrombosis Outcomes Study (CANTOS). *European Heart Journal*.

[93] Ismail F., Baetzner C., Heuer W. (2012). 16S rDNA-based metagenomic analysis of human oral plaque microbiota in patients with atherosclerosis and healthy controls. *Indian Journal of Medical Microbiology*.

[94] Fåk F., Tremaroli V., Bergström G., Bäckhed F. (2015). Oral microbiota in patients with atherosclerosis. *Atherosclerosis*.

